# Clinical- and Cost-Effectiveness of a Nurse Led Self-Management Intervention to Reduce Emergency Visits by People with Epilepsy

**DOI:** 10.1371/journal.pone.0090789

**Published:** 2014-03-06

**Authors:** Adam J. Noble, Paul McCrone, Paul T. Seed, Laura H. Goldstein, Leone Ridsdale

**Affiliations:** 1 Department of Psychological Sciences, Institute of Psychology, Health & Society, University of Liverpool/Department of Clinical Neuroscience, Institute of Psychiatry, King's College London, United Kingdom; 2 Health Service and Population Research, Institute of Psychiatry, King's College London, London United Kingdom; 3 Division of Women's Health, King's College London, London, United Kingdom; 4 Department of Psychology, Institute of Psychiatry, King's College London, London, United Kingdom; 5 Department of Clinical Neuroscience, Institute of Psychiatry, King's College London, London, United Kingdom; University of Puerto Rico, Medical Sciences Campus, Puerto Rico

## Abstract

People with chronic epilepsy (PWE) often make costly, and clinically unnecessary emergency department (ED) visits. Some do it frequently. No studies have examined interventions to reduce them. An intervention delivered by an epilepsy nurse specialist (ENS) might reduce visits. The rationale is it may optimize patients' self-management skills and knowledge of appropriate ED use. We examined such an intervention's clinical- and cost-effectiveness. Eighty-five adults with epilepsy were recruited from three London EDs with similar catchment populations. Forty-one PWE recruited from two EDs received treatment-as-usual (TAU) and formed the comparison group. The remaining 44 PWE were recruited from the ED of a hospital that had implemented a new ENS service for PWE attending ED. These participants formed the intervention group. They were offered 2 one-to-one sessions with an ENS, plus TAU. Participants completed questionnaires on health service use and psychosocial well-being at baseline, 6- and 12-month follow-up. Covariates were identified and adjustments made. Sixty-nine (81%) participants were retained at follow-up. No significant effect of the intervention on ED visits at 12 months or on other outcomes was found. However, due to less time as inpatients, the average service cost for intervention participants over follow-up was less than for TAU participants' (adjusted difference £558, 95% CI, −£2409, £648). Covariates most predictive of subsequent ED visits were patients' baseline feelings of stigmatization due to epilepsy and low confidence in managing epilepsy. The intervention did not lead to a reduction in ED use, but did not cost more, partly because those receiving the intervention had shorter hospital admissions. Our findings on long-term ED predictors clarifies what causes ED use, and suggests that future interventions might focus more on patients' perceptions of stigma and on their confidence in managing epilepsy. If addressed, ED visits might be reduced and efficiency-savings generated.

## Introduction

Epilepsy is a common neurological disorder, with an annual incidence rate of 50–55 per 100,000 person-years [1]. It is also costly. The cost per case in the EU in 2004 was €2,000–11,500 per annum [2]. Studies from around the world show people with epilepsy (PWE) frequently use expensive hospital emergency services [3]–[5]. This helps explain why health costs are so high [6].

Some of the clearest evidence on ED use comes from the U.K., where the prevalence of active epilepsy is approximately 5 cases per 1000 people [1]. Each year, around a fifth of all PWE in the U.K. visit a hospital emergency department (ED) [7], [8]. Approximately one half of these visits result in the patient being admitted [8], [9]. Costs are further increased by PWE visiting ED repeatedly. Around 60% of PWE who attend ED do so on multiple occasions within a 12-month period [10]. The estimated cost of providing emergency care for epilepsy in the U.K. in 2010/11 was £47 million [11], [12].

In addition to being costly, ED visits by PWE are often clinically unnecessary. The majority of ED visits are by those with known, rather than new epilepsy [7], [9] and one of their commonest presentations is that of an uncomplicated seizure [9]. In such instances, emergency medical care is not recommended [13]. Evidence from the U.K.'s National Audit of Seizure Management in Hospitals (NASH) also suggests that ED use has little added value for the care of PWE [7].

What may be important to reducing ED visits by PWE is their ability to self-manage their epilepsy. Coping with life in the context of having epilepsy requires PWE to accept their diagnoses and adopt self-management behaviors to prevent seizures and manage consequences. This includes taking anti-epileptic drugs (AEDs), identifying and managing seizure triggers, implementing precautions to minimize risk, telling others what to do when a seizure occurs, and learning what to do during recovery. Evidence, mainly from cross-sectional surveys, suggests ED visits by PWE are often associated with a failure to master such tasks [10], [14], [15].

What intervention could improve the self-management skills of PWE who visit ED and who should deliver it is currently unknown. An educational intervention aiming to promote self-management skills, delivered by epilepsy nurse specialists (ENS) to PWE, is one possibility. Case-series suggest nurse interventions may reduce emergency visits [16], [17]. Furthermore, trials testing ENS interventions in the wider epilepsy population shows they can lead to improvements in domains potentially relevant to ED use, including patients' epilepsy knowledge [18].

In the present study (ISRCTN06469947) we test the hypothesis that compared to treatment-as-usual (TAU) alone, an ENS-led self-management intervention can reduce reattendance at ED and improve well-being. To do this, we recruited PWE who had attended the EDs of three inner London hospitals with similar catchment populations but which had started to implement different care pathways for PWE. One hospital offered PWE attending their ED access to a new ENS service, whilst the other two hospitals did not. We compared the outcomes of patients and completed an economic evaluation of the services.

## Methods

### Design

Adults attending the EDs for established epilepsy were prospectively recruited (May 2009 – March 2011). Patients recruited from the ED of King's College Hospital (KCH) were offered the new ENS intervention, plus TAU, whilst those recruited from the EDs of St. Thomas' Hospital (STH) and University Hospital Lewisham (UHL) were offered TAU. We have previously described how attendances were identified and participants' representativeness [10].

### Recruitment sites

Together, the three EDs serve one million residents in the London boroughs of Southwark, Lambeth and Lewisham where epilepsy prevalence in adults is 0.51% [19]. The EDs and the populations they predominantly serve, are similar, making comparison of their patients' outcomes reasonable [19]–[21].

### Inclusion criteria

Patients were eligible to participate if they: were aged ≥18; had a documented diagnosis of epilepsy for ≥1 year; could independently complete questionnaires; had no life-threatening or serious co-morbidity; had not seen an ENS in the prior year; had not been referred by ED to Neurology for outpatient care; and resided within Lambeth, Southwark, or Lewisham.

### Ethics Statement

The Joint South London and Maudsley and the Institute of Psychiatry NHS Research Ethics Committee approved the study (08/H0807/86). All participants provided written informed consent.

### Treatment groups

#### ENS intervention (plus TAU) group

The ENS intervention consisted of those attending KCH ED being offered 2 one-to-one sessions delivered on an outpatient basis at KCH. Initial sessions were to last 45–60 minutes and the second 30 minutes. The intervention was planned to be responsive to a patient's individual needs, so the number of sessions completed was permitted to vary. The intervention was informed by the premise that PWE are responsible for their epilepsy's day-to-day management. As such, the ENS was to provide PWE with the knowledge, support and skills to mitigate disability and improve outcome [22]. Table 1 provides details.

**Table 1:** Details of the Epilepsy Nurse-Specialist (ENS) led self-management intervention.

Aspect of intervention	Details
*Premise*	• PWE, as opposed to medical care providers, are responsible for their day-to-day epilepsy management. As such, PWE need the knowledge, support and skills to mitigate disability and improve outcome
	• Aimed to reduce ED visits by optimizing patients' self-management skills and knowledge of appropriate ED use.
*Content*	• Two one-to-one sessions with an ENS.
	• To guide the intervention's delivery and record information given and actions taken by the ENS during sessions, a comprehensive checklist was developed (available from authors on request).
	• Intervention started with the ENS reviewing the patient's epilepsy and checking that the AED(s) and dosing the patient reported taking was consistent with prescription.
	• Information provision formed large component. The areas on which information could be provided included: epilepsy's causes; seizure first aid; the role and mechanisms of AEDs; the importance of adherence and the taking of the same brand; prescription charges; about what to do if a dose is missed; seizure triggers; safety in the home; legal rights of, and benefits available for, PWE epilepsy; and the contact details of support organisations.
	• The ENS informed patients about the names of their seizures and syndrome and having reviewed their existing medical records, probable cause.
	• With regards advice concerning seizure first aid, the ENS the informed the patient what should and should not be done when a seizure occurs and, as a permanent record, provided the patient with an information pamphlet on first aid management of seizures developed by the U.K.'s National Society for Epilepsy [14]. As per these guidelines, participants were informed that when a person with established epilepsy has an epileptic seizure there is usually no need to call an ambulance. For such PWE, emergency medical care was recommended only when any of the following applied: (i) they had sustained a significant injury; (iii) had trouble breathing after the seizure had stopped; (iv) when one seizure immediately followed another with no recovery in between; (v) when the seizure lasted two minutes longer than was usual for them; or the seizure lasts for more than five minutes and it was not known how long their seizures usually last.
	• The ENS developed personalized care plans with the patient, helped them set goals (e.g., to socialize more, be comfortable talking about epilepsy, and less fearful about seizures), evaluated progress and provided the patient with the opportunity to ask questions.
	• The ENS could make referrals, tailored to the patients' requirements, by normal pathways to other services (e.g., counselling, social services, and emergency rescue medication clinic). Any advice given and actions taken were communicated to the patient's primary care doctor.
	• At appointments, participants had direct access to either of two “Expert Patients” in the waiting room who were trained by the U.K.'s National Society for Epilepsy, and were invited to join a service users' group.
	• Carers accompanied patients when PWE requested this.
*Those delivering intervention*	• Sessions were delivered by either one of the two ENSs based at KCH.
	• Before the implementation of this new service, for reasons of limited service capacity, the ENSs only accepted direct referrals from neurologists and neurosurgeons. They ran clinics, but, as was the case this new service, did not independently prescribe AEDs. One had 8 years of experience working as an ENS and the other 10.

**Notes**→AED =  antiepileptic drug; ED =  emergency department; ENS =  Epilepsy Nurse Specialist; KCH = King's College Hospital; PWE =  people with epilepsy.

#### TAU group

Following recruitment, no restrictions were placed on the services TAU participants could access. In the U.K., there is no accepted care for those with established epilepsy who have visited an ED. All PWE are though expected to have a medical review of their epilepsy at least yearly by a generalist or specialist. When seizures are not controlled or treatment fails, it is expected that a patient will be referred to secondary or tertiary services [23]. The U.K.'s NASH showed EDs initiate referral to neurology for only a third of PWE attending ED [9]. At the time of the study, no ENS services were part of TAU at STH or UHL.

### Baseline and outcome measures

Participants were assessed at three points using validated questionnaires: baseline (assessment 1), 6- (assessment 2) and 12-months post recruitment (assessment 3). For assessments 1 and 2, measures were completed face-to-face. Assessment 3 was completed by post.

#### Clinical outcomes

These included self-report measures for epilepsy-related ED use [24] and patient well-being [25]. The latter included epilepsy-specific quality of life (QoL) [31], seizure frequency [26]–[28], medication management skills [29], psychological distress [30], felt stigma [31], confidence in managing epilepsy (i.e., mastery) [32] and epilepsy knowledge [33]. Table S1 details the questionnaires and gives example items.

#### Cost-effectiveness

This evaluation primarily adopted a healthcare perspective with the cost of providing care for participants in the two treatment groups over follow-up being compared. The cost of lost employment was included in further analyses.

To ascertain health-care costs, participants reported health service use on the Client Service Receipt Inventory (CSRI) [24] at baseline and at follow-up assessments. The CSRI referred to the previous 12 months at assessment 1 and the previous 6 months at assessments 2 and 3. Table S1 describes the CSRI. Costs were calculated by combining service use data with appropriate national unit costs [34]. The epilepsy nurse cost was estimated at £50 per hour. This takes into account salaries, overheads, capital costs, training, and the ratio of direct to indirect contact time.

To promote the comparison of interventions between studies, a generic outcome measure should be incorporated into economic evaluations [35]. We used the most common method, the quality-adjusted life year (QALY). A QALY adjusts time spent in a health state by a utility score anchored by 1 (full health) and 0 (death). The European Quality of Life-5 Dimensions (EQ-5D) [36], the measure favoured by decision makers in England and other countries, combined with U.K. weights [37], was used to generate utility scores at baseline and follow-up. Area under the curve methods were then used to estimate the QALYs gained during the follow-up period.

### Sample size

The primary outcome measure was ED visits. No evidence existed on what sort of effect the intervention might have on these. Jacoby et al. [4] found 27% of PWE in the U.K. with uncontrolled epilepsy make at least one ED visit per year. We hypothesized that the ENS intervention might reduce re-attendance to 7% (Rate Ratio 0.26), partly by more effective self-management, partly by more frequent and appropriate use of non-emergency services. Following Parmar and Machin's [38] formulae, two groups of 60 would give 80% power to detect such a difference. We planned to recruit 160 participants into our study in order to allow for 25% loss to follow-up. It was considered that a reduction of 20% (number needed to treat 5) would be needed if the intervention was to gain general acceptability.

### Statistical analyses

#### Treatment group equivalence and care received

Descriptive statistics describe the characteristics of those recruited into the treatment groups, those retained at follow-up and the epilepsy care they received. Logistic regression tested for the significance of any differences between the groups. Odds-ratios (OR) and 95% confidence intervals (CI) are presented.

#### Effect of ENS intervention on clinical outcomes

The primary outcome was the number of ED visits participants reported making over the 6 months preceding assessment 3. Secondary measures were the number of visits made over the 6 months preceding assessment 2 and patient well-being at assessments 2 and 3.

The analyses of the effect of the intervention on each of the outcome measures were performed using an intention-to-treat (ITT) approach. To further understand any treatment effects, efficacy based comparisons were also completed. These included in the intervention group only those participants who had received at least one intervention session. For all analyses, participants from the two non-intervention sites were pooled to form one single TAU group.

#### Effect on ED use

To compare the ENS intervention's effect on ED use to TAU alone, negative binomial regression (NBR) was used to determine if treatment group predicted ED visits over follow-up. Over dispersion of ED visits meant NBR, with robust standard errors, was appropriate [39]. A P-value of <0.05 was considered statistically significant.

Unadjusted NBR analyses were first completed. However, to account for imbalances between treatment groups in baseline characteristics, we also completed adjusted NBR. This involved first examining the association between scores on each baseline measure and ED visits at assessments 2 and 3. Covariates with a marginal association (P<0.10) were then included in the applicable adjusted analyses. The adjustments made for each model are indicated in the table notes.

Treatment effect estimates are presented as incidence rate-ratios (IRRs), with 95% CIs. IRRs less than 1 represent a lower visit rate in the ENS treatment group relative to TAU, whilst IRRs greater than 1, indicate a higher rate.

#### Effect on patient well being

Scores on the measures of patient well-being were treated as continuous and linear regression, with robust standard errors, tested for treatment effects. Results from unadjusted and adjusted analyses are presented. Treatment effect estimates are presented in the form of unstandardized coefficients.

#### Cost-effectiveness

Total service costs of the two groups at the follow-up assessment points were compared using linear regression with adjustment for baseline cost. Bootstrapped 95% CIs were generated around the regression coefficient representing the cost difference.

Healthcare costs were combined with QALY data in the form of an incremental cost-effectiveness ratio (ICER), calculated by dividing the incremental costs for the ENS treatment group compared to the comparison group by the incremental QALY gain. To address uncertainty around the ICER, we generated 1000 resamples using bootstrapping with replacement and calculated cost and outcome differences for each resample. These 1000 cost-outcome pairs were plotted on to a cost-effectiveness plane.

All analyses were performed using STATA 11 (Stata Corporation, College Station, TX, U.S.A.)

## Results

### Participants

#### Recruitment and treatment group equivalence at baseline

**Figure 1:**
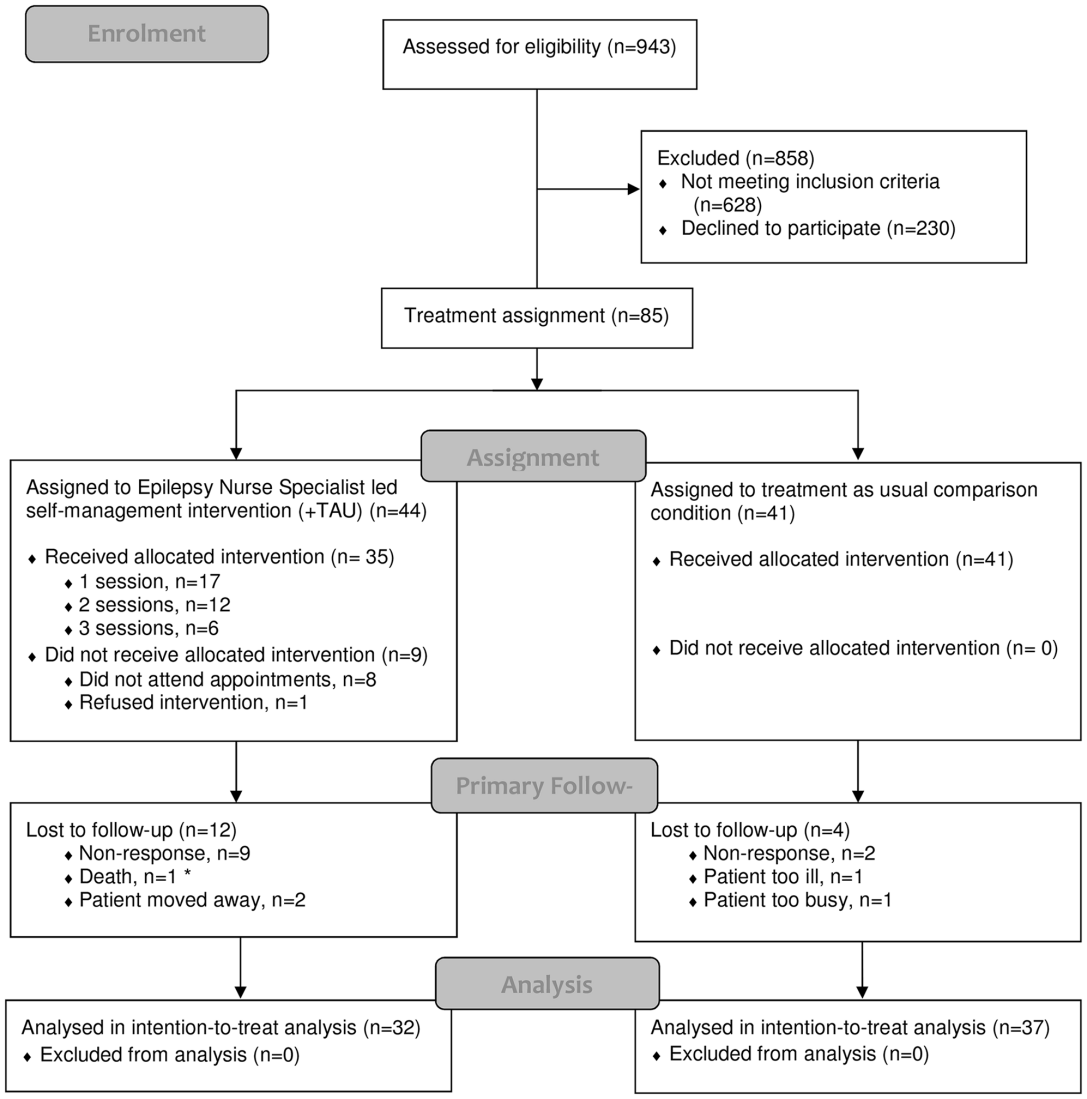
Flow diagram of participant recruitment, treatment allocation and retention. Note to Figure 1: * Participant died of sudden unexplained death in epilepsy. This patient was allocated to the intervention study arm, but failed to attend all offers of appointments prior to death.

Three hundred and fifteen eligible PWE were identified and 85 agreed to participate. Figure 1 depicts recruitment and retention. Mean participant age was 41 (SD = 16) and 53% were male. Median years since diagnosis was 11 (interquartile range [IQR] = 6–28).

Forty-four participants formed the ENS treatment group and 41 the TAU comparison group. At assessment 1 (baseline), the two groups were similar (Table 2). The TAU group did, however, report having experienced more seizures in the previous year. Their median seizure number was 10 (IQR =  1.2–4.5) compared to 5.5 (IQR = 1.0–3.0) for participants in the ENS treatment group.

**Table 2:** Baseline characteristics of study participants according to treatment group and assessment.

Baseline measure (n/ %)	Treatment groups at baseline		
	*TAU group (n = 41)*	*ENS group (n = 44)*	*OR (95% CI)*
**Age at baseline**			
18–24	6 (14.6)	8 (18.2)	1.00 Reference
25–34	8 (19.5)	12 (27.3)	1.55 (0.56, 4.31)
35–45	7 (17.1)	7 (15.9)	0.92 (0.29, 2.91)
46–53	12 (29.3)	8 (18.2)	0.54 (0.19, 1.50)
54–89	8 (19.5)	9 (20.5)	1.06 (0.36, 3.09)
**Gender**			
Male	22 (53.7)	24 (54.5)	1.00 Reference
Female	19 (46.3)	20 (45.5)	0.97 (0.41, 2.28)
**Ethnicity**			
Other	17 (41.5)	17 (38.6)	1.00 Reference
White British	24 (58.5)	27 (61.4)	0.89 (0.37, 2.13)
**Years of formal education**			
10 Least educated	2 (4.9)	1 (2.3)	1.00 Reference
11	24 (58.5)	19 (43.2)	0.54 (0.23, 1.28)
12	2 (4.9)	2 (4.5)	0.93 (0.12, 7.00
13–15.5	6 (14.6)	10 (22.7)	1.72 (0.56, 5.28)
16–24 Most educated	7 (17.1)	12 (27.3)	1.82 (0.63, 5.24)
**Deprivation score**			
13.97–22.70 Least deprived	5 (12.2)	12 (27.3)	1.00 Reference
23.36–28.98	9 (22.0)	8 (18.2)	0.79 (0.27, 2.31)
29.75–33.46	7 (17.1)	10 (22.7)	1.43 (0.48, 4.22)
33.56–38.31	11 (26.8)	7 (15.9)	0.52 (0.18, 1.50)
38.76–47.46 Most deprived	9 (22.0)	7 (15.9)	0.67 (0.22, 2.02)
**Co-morbidity**			
None	23 (56.1)	20 (45.5)	1.00 Reference
Psychiatric and/or medical	18 (43.9)	24 (54.5)	1.53 (0.65, 3.63)
**Years epilepsy diagnosed**			
2–4	5 (12.2)	10 (22.7)	1.00 Reference
5–8	9 (22.0)	7 (15.9)	0.67 (0.22, 2.02)
9–15	7 (17.1)	13 (29.5)	2.04 (0.72, 5.80)
16–34	9 (22.0)	8 (18.2)	0.79 (0.27, 2.31)
35–67	11 (26.8)	6 (13.6)	0.43 (0.14, 1.31)
**ED visits prior 12 months**			
1	15 (36.6)	18 (40.9)	1.00 Reference
2	12 (29.3)	10 (22.7)	0.71 (0.27, 1.90)
3–4	3 (7.3)	8 (18.2)	2.82 (0.69, 11.55)
5–25	11 (26.8)	8 (18.2)	0.61 (0.22, 1.71)
**Seizures prior 12 months**			
1–2	7 (17.1)	10 (22.7)	1.00 Reference
3–5	6 (14.6)	12 (27.3)	2.19 (0.73, 6.56)
6–9	6 (14.6)	8 (18.2)	1.30 (0.41, 4.15)
** 10 or more**	**22 (53.7)**	**14 (31.8)**	**0.40 (0.17, 0.98)**
**Seizure severity score**			
0–5 Least severe	13 (32.5)	20 (45.5)	1.00 Reference
7.5–50	10 (25)	6 (13.6)	0.47 (0.15, 1.46)
52.5–67.5	9 (22.5)	8 (18.2)	0.77 (0.26, 2.24)
70–90 Most severe	8 (20)	10 (22.7)	1.18 (0.41, 3.38)
**Seizure onset**			
Generalized or unknown	19 (46.3)	17 (38.6)	1.00 Reference
Focal	22 (53.7)	27 (61.4)	1.37 (0.58, 3.27)
**AEDS prescribed**			
0	1 (2.4)	2 (4.5)	1.00 Reference
1	18 (43.9)	26 (59.1)	1.85 (0.78, 4.39)
2	16 (39.0)	13 (29.5)	0.66 (0.27, 1.62)
3–5	6 (14.6)	3 (6.8)	0.43 (0.10, 1.85)
**Depression score**			
0–1 Least symptoms	11 (26.8)	2 (4.5)	1.00 Reference
2–3	11 (26.8)	26 (59.1)	0.70 (0.26, 1.93)
4–5	4 (9.8)	0 (0.0)	2.72 (0.77, 9.56)
6–7	7 (17.1)	13 (29.5)	1.43 (0.48, 4.22)
8–19 Most symptoms	8 (19.5)	3 (16.8)	1.38 (0.49, 3.88)
**Anxiety score**			
0–4 Least symptoms	7 (17.1)	7 (15.9)	1.00 Reference
5–7	10 (24.4)	12 (27.3)	1.16 (0.44, 3.10)
8–9	8 (19.5)	6 (13.6)	0.65 (0.20, 2.09)
10–12	9 (22.0)	10 (22.7)	1.05 (0.37, 2.92)
13–19 Most symptoms	7 (17.1)	9 (20.5)	1.25 (0.42, 3.76)
**QOL score**			
13–18 Highest QoL	9 (22.0)	7 (15.9)	1.00 Reference
19–23	7 (17.1)	11 (25.0)	1.62 (0.56, 4.71)
24–26	6 (14.6)	8 (18.2)	1.30 (0.41, 4.14
27–33	11 (26.8)	8 (18.2)	0.61 (0.22, 1.71)
34–36 Lowest QoL	8 (19.5)	10 (22.7)	1.21 (0.42, 3.47)
**Felt stigma score**			
0 Least stigma	12 (29.3)	15 (34.1)	1.00 Reference
1–2	9 (22.0)	10 (22.7)	1.05 (0.37, 2.92)
3–4	8 (19.5)	13 (29.5)	1.73 (0.63, 4.77)
5–9 Most stigma	12 (29.3)	6 (13.6)	0.38 (0.13, 1.15)
**Medication management**			
13–39 Lowest skills	5 (12.5)	11 (25.0)	1.00 Reference
40–44	6 (15.0)	10 (22.7)	1.90 (0.65, 5.54)
45–46	9 (22.5)	10 (22.7)	1.18 (0.44, 3.16)
47–48	8 (20.0)	7 (15.9)	0.93 (0.32, 2.72)
49–50 Highest skills	12 (30.0)	6 (13.6)	0.48 (0.16, 1.41)
**Satisfaction info**			
1–4 Least satisfied	7 (17.5)	7 (16.3)	1.00 Reference
5–7	8 (20.0)	10 (23.3)	1.21 (0.42, 3.48)
8–9	6 (15.0)	9 (20.9)	1.50 (0.48, 4.71)
10–11	8 (20.0)	10 (23.3)	1.21 (0.42, 3.48)
12–17 Most satisfied	11 (27.5)	7 (16.3)	0.51 (0.18, 1.50)
**Social knowledge**			
8–12 Lowest knowledge	10 (24.4)	5 (11.4)	1.00 Reference
13–14	13 (31.7)	13 (29.5)	0.90 (0.36, 2.29)
15–15	9 (22.0)	13 (29.5)	1.49 (0.56, 4.01)
16–20 Highest knowledge	9 (22.0)	13 (29.5)	1.49 (0.56, 4.01)
**Medical knowledge**			
15–21 Lowest knowledge	11 (26.8)	7 (15.9)	1.00 Reference
22–24	9 (22.0)	8 (18.2)	0.79 (0.27, 2.31)
25–26	7 (17.1)	8 (18.2)	1.08 (0.35, 3.32)
27–28	7 (17.1)	11 (25.0)	1.62 (0.56, 4.71)
29–32 Highest knowledge	7 (17.1)	10 (22.7)	1.43 (0.48, 4.22)
**Mastery**			
6–12 Lowest confidence	10 (24.4)	8 (18.2)	1.00 Reference
13–14	8 (19.5)	11 (25.0)	1.38 (0.49, 3.88)
15–15	5 (12.2)	8 (18.2)	1.60 (0.47, 5.40)
16–17	8 (19.5)	10 (22.7)	1.21 (0.42, 3.47)
18–21 Highest confidence	10 (24.4)	7 (15.9)	0.59 (0.20, 1.73)

**Notes**→AED =  antiepileptic drug; CI =  Confidence interval; ED =  Emergency department; ENS =  Epilepsy Nurse Specialist; OR =  Odds-ratio; Primary care QoF 8 score =  Quality and Outcomes Framework; percentage of people with epilepsy (aged ≥16) prescribed AEDs in the local population who were seizure free in the previous 12 months as recorded by primary care medical practices in England in 2009/10; QoL =  Quality of Life; TAU =  Treatment as usual.

P<0.10 shown in **bold**; Logistic regression used.

#### Retention at follow-up

Sixty-nine (81%) participants were retained at assessments 2 and 3. This included 37 (90%) participants from the TAU group and 32 (73%) participants from the ENS group. It is on these participants that ITT analyses were based.

Those lost to follow-up differed from those retained in their baseline characteristics. This, together with unequal loss to follow-up between treatment groups, further imbalanced the characteristics of the two groups (Table S2). As well as the ENS group still having had fewer seizures, at baseline it now comprised fewer participants who had felt highly stigmatized by epilepsy. At the same time, fewer TAU group participants had a co-morbid condition.

#### Receipt of the ENS intervention

Over the entire 12-month follow-up period, 35 (80%) of the 44 recruited participants offered the intervention attended. It is this subgroup of participants who formed the treatment group for the purposes of the efficacy based analyses.

Of the 35 participants, 17 (39%) attended 1 ENS session, 12 (27%) 2 sessions and 6 (14%) 3 sessions. The first session took place on average 5 weeks following recruitment, the second 24 weeks and the third 38 weeks later. No baseline characteristic was found to significantly predict whether an intervention participant received at least one ENS session or not (all P<0.05).

### Effect of ENS intervention on clinical outcomes

#### Unadjusted analyses of effect on ED use

Table 3 presents the pattern of ED use reported by the two groups. The rate of ED visits reported by the ENS treatment group at assessment 3 was 55% lower than the TAU comparison groups (Table 4). This difference was though not statistically significant (P = 0.113), with group not significantly predicting ED use in the ITT analyses (Wald *X^2^* (1) = 2.52, P = 0.1127). No significant difference was also found in the rate of visits reported by the groups at assessment 2 according to the ITT analyses.

**Table 3:** Emergency department visits for epilepsy reported at baseline and at follow-up assessments.

	Emergency department visits *n (%)*			
	*0*	*1*	*2–3*	*≥4*
**Baseline (visits during prior 12 months)**				
TAU group (n = 41)	0	15 (36.6)	13 (31.7)	13 (31.7)
ENS group (n = 44)	0	18 (40.9)	15 (34.1)	11 (25.0)
**Assessment 2 (visits during prior 6 months)**				
TAU group (n = 37)	23 (62.2)	6 (16.2)	4 (10.8)	4 (10.8)
ENS group (n = 32)	13 (40.6)	10 (31.3)	7 (21.9)	2 (6.3)
**Assessment 3 (visits during prior 6 months)**				
TAU group (n = 37)	23 (62.2)	4 (10.8)	6 (16.2)	4 (10.8)
ENS group (n = 32)	22 (68.8)	3 (9.4)	6 (18.8)	1 (3.1)

**Notes**→Frequency of emergency department visits was over-dispersed at both 6- (M 1.12< variance 4.34; *X^2^* (1) = 50.93, P<0.001) and 12-month follow-up (M 1.13 variance 7.65; *X^2^* (1) = 111.65, P<0.001); ENS =  Epilepsy Nurse Specialist; TAU =  Treatment as usual.

**Table 4:** Intention-to-treat analysis comparing treatment groups on primary and secondary outcome measures.

Outcome measure	Assessment 2 (n = 69)		Assessment 3 (n = 69)	
	*Unadjusted*	*Adjusted*	*Unadjusted*	*Adjusted*
	*IRR/ Coefficient (95% CI)*	*IRR/ Coefficient (95% CI)*	*IRR/ Coefficient (95% CI)*	*IRR/ Coefficient (95% CI)*
**Primary outcome measure**				
Emergency department visits	1.07 (0.45, 2.54)	1.75 (0.93, 3.28)	0.45 (0.17, 1.20)	1.92 (0.68, 5.41)
**Secondary outcome measure**				
Quality of Life (higher = poorer quality)	1.29 (−2.35, 4.94)	0.98 (−1.40, 3.36)	2.65 (−1.06, 6.37)	3.20 (−0.59, 6.98)
Seizure frequency (higher = more seizures)	−0.27 (−2.30, 1.74)	0.51 (−1.10, 2.12)	−0.27 (−2.19, 1.65)	0.58 (−0.97, 2.13)
Anxiety (higher = more symptoms)	−0.41 (−2.64, 1.83)	−1.01 (−2.56, 0.55)	−1.04 (−3.29, 1.20)	−1.72 (−3.70, 0.25)
Depression (higher = more symptoms)	0.25 (−1.68, 2.17)	−0.67 (−1.94, 0.59)	0.18 (−1.72, 2.08)	−0.03 (−1.88, 1.82)
Medication management skills (higher = better skills)	**−2.70 (−4.63, 0.77)**	−1.28 (−2.94, 0.38)	−1.26 (−5.50, 2.97)	1.85 (−1.47, 44.99)
Mastery (higher = greater confidence)	−0.46 (−2.14, 1.21)	−0.80 (−2.23, 0.62)	0.32 (−1.33, 1.98)	−0.49 (−2.10, 1.12)
Epilepsy social knowledge (higher = more knowledgeable)	0.04 (−1.18, 1.25)	−0.86 (−1.82, 0.11)	-	-
Epilepsy medical knowledge (higher = more**knowledgeable)	0.32 (−1.54, 2.17)	−0.94 (−2.22, 0.34)	-	-
Felt stigma (higher = more stigmatization)	−0.69 (−2.03, 0.64)	0.01 (−0.85, 0.85)	-	-
Satisfaction with medication information** (higher = more satisfied)	0.31 (−1.43, 0.82)	−0.16 (−2.40, 2.08)	-	-

**Notes**→IRR =  incidence rate-ratio; CI =  confidence interval; AED  =  antiepileptic drug; ED Emergency Departments.

P<0.05 shown in **bold**; Negative binomial regression used for outcome measure emergency visits and linear regression used for all remaining measures.

IRRs less than 1 here represent a lower visit rate in the ENS intervention group relative to TAU group, whilst IRRs greater than 1, indicate a higher rate.

For secondary outcome measures, positive coefficients here indicate an increase in the score on the outcome variable associated with receiving the ENS led self-management intervention, whilst a negative coefficient the opposite.

Adjustments were made for baseline variables related to outcome at P<0.10:

Emergency department (ED) visits: Baseline Seizure frequency (assessment 3) ED visits (assessments 2, 3), Seizure severity (3), AED number (2,3), Depression (2,3), Anxiety (2,3), Quality of Life (QoL) (3), Felt stigma (3), Medical knowledge (3), Mastery (2,3). Number of covariates in final assessment 2 model = 5; Number of variables in final assessment 3 model = 10.

QoL: Baseline Seizure frequency (2,3), ED visits (2,3), AED number (2), Depression (2,3), Anxiety (2, 3), QoL (2,3), Stigma (2,3), Satisfaction medication information (2), Social knowledge (3), Medical knowledge (3), Mastery (2,3). Number of covariates in final assessment 2 model = 9; Number of covariates in final assessment 3 model = 9.

Seizure frequency: Baseline Seizure frequency (2,3), Primary care seizure-free rate (QOF score 8) (3), Gender (2), ED visits (2,3), Seizure severity (2), AED number (2,3), Depression (2,3), Anxiety (2,3), QoL (2,3), Felt stigma (2,3), Medication management (2), Social knowledge (3), Mastery (2,3). Number of covariates in final assessment 2 model = 11; Number of covariates in final assessment 3 model = 10.

Anxiety: Baseline Seizure frequency (3), ED visits (2,3), AED number (2), Depression (2,3), Anxiety (2,3), QoL (2,3), Felt stigma (2,3), Social knowledge (3), Mastery (2,3). Number of covariates in final assessment 2 model = 7; Number of covariates in final assessment 3 model = 8.

Depression: Baseline Age (3), Education (3), Deprivation (3), ED visits (2,3), Depression (2,3), Anxiety (2,3), QoL (2,3), Felt stigma (2,3), Social knowledge (3), Medical knowledge (3), Satisfaction with medication information (2), Mastery (2,3). Number of covariates in final assessment 2 model = 7; Number of covariates in final assessment 3 model =  11.

Medication Management Skills: Baseline Age (2), Sex (2), Epilepsy duration (2), AED number (3), Depression (3), Medication Management (3), Medical knowledge (3). Number of covariates in adjusted assessment 2 model =  3; Number of covariates in final assessment 3 model = 4.

Mastery: Baseline Seizure frequency (2,3), Gender (2), Ethnicity (3), Deprivation (3), ED visits (2,3), Seizure severity (2,3), AED number (2,3), Depression (2,3), Anxiety (2,3), QoL (2,3), Felt stigma (2,3), Social knowledge (3), Medical knowledge (3), Mastery (2,3). Number of covariates in final assessment 2 model = 10; Number of covariates in final assessment 3 model = 13.

Epilepsy social knowledge: Baseline Age, Education, Deprivation, Medication management skills, Social knowledge, Medical knowledge. Number of covariates in final assessment model = 6.

Epilepsy medical knowledge: Baseline Age, Education, Deprivation, ED visits, Depression, Anxiety, Felt stigma, Social knowledge, Medical knowledge, Mastery. Number of covariates in final assessment model = 10.

Felt stigma: Baseline Seizure frequency, Ethnicity, Deprivation, ED visits, Seizure severity, AED number, Depression, QoL, Felt stigma, Mastery. Number of covariates in final assessment model = 10.

Satisfaction with medication information: Baseline Primary care QOF 8 score, Deprivation, ED visits, Depression, Anxiety, QoL, Felt stigma, Satisfaction with medication information, Medical knowledge, Mastery. Number of covariates in final assessment model = 10.

No significant effect of the ENS intervention on subsequent ED visits was found when analyses were restricted to include in the ENS group only those participants who had received at least one intervention session (Table S3).

#### Adjusted analyses of the effect on ED use

The baseline variables identified by univariate screening as predictive of greater ED visits following recruitment were, in descending order of importance, lower confidence in managing epilepsy (less mastery), higher number of prescribed AEDs, more felt stigma, higher number of baseline ED visits, greater seizure frequency, and higher levels of depression and anxiety.

Including these covariates in the regression models for ED visits resulted in the models now significantly predicting the ED visits participants reported having made both 6- (Wald *X^2^* (6) =  103.30, P<0.0001) and 12-months following recruitment (Wald *X^2^* (11) =  140.90, P<0.0001). Treatment group, however, remained a non-significant predictor when the data was analysed on both an ITT (Table 4) and an efficacy basis (Table S3). In these multivariate analyses, it was greater felt stigma and less confidence in managing epilepsy which emerged as significant predictors of ED visits at assessment 3 (Table 5).

**Table 5:** Association between baseline variables and emergency department visits made by participants over follow-up.

Baseline measure	Assessment 2		Assessment 3	
	*Unadjusted IRR (95% CI)*	*Adjusted IRR (95% CI)*	*Unadjusted IRR (95% CI)*	*Adjusted IRR (95% CI)*
Gender (0 = female; 1 = male)	0.69 (0.31, 1.55)	-	0.97 (0.30, 3.12)	-
Age (years)	0.99 (0.98, 1.02)	-	1.01 (0.98, 1.02)	-
Ethnicity (0 = White British; 1 = other)	1.30 (0.52, 3.25)	-	2.40 (0.84, 6.87)	-
Education (years)	0.92 (0.80, 1.06)	-	0.94 (0.82, 1.09)	-
Deprivation (higher = more deprivation)	0.97 (0.93, 1.01)	-	0.99 (0.93, 1.06)	-
Co-morbidity (0 = none; 1 = present)	0.94 (0.40, 2.22)	-	1.32 (0.45, 3.83)	-
Duration of epilepsy (years)	0.99 (0.97, 1.02)	-	0.99 (0.97, 1.03)	-
Emergency visits in prior 12 months	**1.14 (1.10, 1.19)**	**1.14 (1.03, 1.26)**	**1.19 (1.10, 1.29)**	1.05 (0.92, 1.20)
Quality of life (higher = poor quality of life)	1.04 (0.99, 1.10)	-	**1.09 (1.02, 1.16)**	0.93 (0.86, 1.01)
Seizure frequency	1.09 (0.97, 1.23)	-	**1.19 (1.03, 1.38)**	0.91 (0.80, 1.02)
Primary care QoF 8 score (higher = more seizure free)	1.01 (0.98, 1.03)	-	0.98 (0.96, 1.01)	-
Seizure severity (higher = more severe)	1.01 (0.99, 1.02)	-	**1.03 (1.01, 1.04)**	1.02 (0.99, 1.03)
Seizure localization (0 = Generalized or**unknown, 1 = Focal)	0.55 (0.24, 1.24)	-	0.66 (0.23, 1.96)	-
Number of AEDs prescribed	**1.56 (1.13, 2.15)**	0.98 (0.68, 1.41)	**1.69 (1.18, 2.44)**	1.43 (0.83, 2.47)
Depression (higher = more symptoms)	**1.12 (1.04, 1.20)**	0.99 (0.88, 1.12)	**1.16 (1.07, 1.25)**	0.99 (0.87, 1.14)
Anxiety (higher = more symptoms)	**1.13 (1.04, 1.23)**	1.02 (0.94, 1.11)	1.10 (0.99, 1.22)	
Felt stigma (higher = more felt stigma)	**1.14 (1.01, 1.30)**	0.97 (0.82, 1.10)	**1.42 (1.19, 1.69)**	**1.32 (1.11, 1.56)**
Medication Management Skills (higher = better skills)	0.97 (0.92, 1.03)	-	1.01 (0.96, 1.06)	-
Satisfaction with information (higher = **increased satisfaction)	0.93 (0.83, 1.04)	-	0.89 (0.77, 1.04)	-
Medical knowledge (higher = more knowledge)	0.95 (0.87, 1.03)	-	0.92 (0.83, 1.02)	-
Social knowledge (higher = more knowledge)	0.88 (0.71, 1.09)	-	0.80 (0.60, 1.06)	-
Mastery (higher = more confidence in managing epilepsy)	**0.85 (0.78, 0.93)**	0.95 (0.87, 1.04)	**0.77 (0.70, 0.84)**	**0.86 (0.77, 0.96)**
**Model summary**		***X*** **^2^ (6) = 43.69, P<0.0001**		***X*** **^2^ (8) = 120.91, P<0.0001**

**Notes**→IRR =  incidence rate-ratio; CI =  confidence interval; AEDs =  antiepileptic drugs; Primary care QoF 8 score =  Quality and Outcomes Framework; percentage of people with epilepsy (aged ≥16) prescribed AEDs in the local population who were seizure free in the previous 12 months as recorded by primary care medical practices in England in 2009/10.

P<0.05 shown in **bold**; Negative binomial regression used.

#### Effect on patient well-being

No significant effect of the ENS intervention was found on any of the measures of patient well-being at the primary, 12-month outcome assessment, both when analysed on an ITT basis (Table 4) and when analysed using an efficacy based approach (Table S3).

### Cost effectiveness

The mean total service cost over the entire follow-up period was £2948 for the TAU treatment group and £2202 for the ENS treatment group (Table 6). The average adjusted difference in service costs was £558 (95% bootstrapped CI –£2409 to £648) less for intervention group participants than for TAU participants. This was accounted for by differences between the groups in their pattern of service use which were most pronounced during the initial 6 months of follow-up.

**Table 6:** Use and cost of health services and lost employment cost for participants according to treatment group.

	Assessment 2 (n = 69)						Assessment 3 (n = 69)					
Service	TAU group			ENS group			TAU group			ENS group		
	*N (%)*	*Mean (SD) contacts*	*Mean (SD) cost*	*N (%)*	*Mean (SD) contacts*	*Mean (SD) cost*	*N (%)*	*Mean (SD) contacts*	*Mean (SD) cost*	*N (%)*	*Mean (SD) contacts*	*Mean (SD) cost*
ED	14 (38)	2.9 (3.4)	53 (122)	17 (57)	1.7 (1.3)	44 (61)	14 (38)	4.0 (5.0)	74 (175)	10 (31)	2.2 (1.2)	34 (60)
Inpatient stays	5 (15)	11.6 (21.5)	633 (3320)	4 (13)	2.7 (2.1)	101 (384)	8 (22)	3.5 (4.8)	306 (1036)	2 (6)	4.5 (2.1)	114 (473)
ED short-stay ward	5 (14)	1.8 (1.3)	144 (451)	11 (37)	1.1 (0.3)	222 (328)	9 (24)	2.3 (1.1)	337 (678)	6 (19)	2.0 (1.5)	222 (598)
Neurology O/P	23 (62)	1.3 (0.6)	119 (114)	21 (66)	1.2 (0.4)	119 (102)	22 (60)	1.4 (0.7)	119 (124)	19 (59)	1.5 (0.6)	133 (131)
Other O/P	17 (46)	2.2 (1.5)	147 (216)	15 (47)	1.5 (0.9)	106 (146)	5 (14)	2.0 (1.2)	40 (118)	14 (44)	1.4 (0.9)	92 (138)
Day care	2 (5)	2.5 (2.1)	12 (62)	3 (9)	1.0 (0.0)	9 (27)	1 (3)	1.0 (−)	4 (24)	6 (19)	2.0 (1.7)	55 (153)
Primary care doctor	27 (73)	3.6 (2.1)	71 (79)	25 (78)	3.6 (2.3)	105 (111)	22 (60)	3.6 (2.2)	92 (141)	23 (72)	4.1 (3.0)	140 (240)
ENS	2 (5)	1.0 (0.0)	2 (7)	27 (84)	1.6 (0.7)	51 (34)	3 (8)	1.3 (0.6)	2 (8)	8 (25)	1.5 (1.1)	11 (31)
Primary care nurse	20 (54)	2.0 (2.1)	8 (14)	7 (23)	1.4 (0.5)	4 (8)	9 (24)	1.9 (1.6)	6 (25)	6 (19)	1.2 (0.4)	2 (5)
Physiotherapist	2 (5)	3.0 (1.4)	3 (16)	1 (3)	2.0 (−)	1 (6)	1 (3)	2.0 (−)	2 (9)	3 (9)	4.7 (1.5)	14 (51)
Social worker	0 (0)	-	0 (0)	6 (19)	3.3 (4.3)	68 (238)	1 (3)	1.0 (−)	3 (17)	3 (9)	2.0 (1.7)	36 (154)
Medication	35 (95)	-	260 (245)	31 (97)	-	230 (204)	35 (95)	-	309 (299)	30 (94)	-	234 (248)
*Total health and social care cost*	-	-	*1461 (3643)*	*-*	*-*	*1065 (781)*	-	-	*1293 (1764)*	*-*	*-*	*1088 (944)*
												
Lost work days	3 (8)	4.7 (2.1)	30 (111)	6 (19)	4.8 (3.9)	73 (197)	8 (22)	8.0 (9.7)	138 (434)	4 (13)	3.3 (2.2)	33 (103)
**Total cost**	**-**	**-**	**1492 (3648)**	**-**	**-**	**1138 (840)**	**-**	**-**	**1431 (1784)**	**-**	**-**	**1121 (927)**

**Notes**→Costs in 2010/11 £s; ENS =  Epilepsy Nurse Specialist; O/P =  outpatient appointment; TAU =  Treatment as usual.

During the initial 6 months, more ENS group participants visited an ED for epilepsy (57%) compared to the TAU group (38%), but for those who did the mean number of visits was less (1.7 vs. 2.9) (Table 5). Also, whilst a similar number of participants from both treatment groups had hospital admissions, these were longer for the TAU group. The costs associated with inpatient care were 527% higher for the TAU group.

The QALY gain over the follow-up period for the ENS treatment group was 0.786 compared to 0.807 for the TAU group. The mean difference, adjusting for baseline differences, was 0.0211, which was not statistically significant (bootstrapped 95% CI, −0.09 to 0.04). Based on the average costs and QALY difference, the ENS intervention resulted in lower costs but fewer QALYs. The ICER was −£558 divided by −0.0211. This means that it costs an extra £26,445 to achieve one extra QALY if the ENS intervention is *not* used.

## Discussion

### Principal findings

Health service planners need cost-effective interventions to reduce unnecessary emergency visits by PWE and resulting admissions. A self-management intervention delivered by an ENS had been proposed as potentially able to reduce visits. We compared its clinical- and cost-effectiveness to TAU alone.

Eighty-percent of the participants offered the intervention in our study attended at least one intervention session. This uptake rate is favourable when compared to trials of nurse interventions in the wider epilepsy population [18], [40]–[44]. The ENS-led intervention did not though lead to a statistically significant benefit in terms of reducing subsequent visits to ED, nor was there any improvement on the secondary measures of patient well-being. However, recruitment into our study was slower than anticipated, and the study finished with 69 participants instead of the planned 120. This meant our study was underpowered to detect the hypothesised effect on ED use and there this consequent ambiguity in some conclusions. The results from our adjusted analyses are, nevertheless, evidence against the possibility of a large reduction in ED visits.

### Potential reasons why the intervention might not lead to a large reduction in ED visits

Firstly, whilst previous evidence had suggested a nurse-led intervention could reduce ED visits [16], [17], this came from studies using weaker methodologies. Studies had compared ED visits in patients before and after receiving such interventions, but did not have a TAU comparison group. All reductions in ED visits were, therefore, attributed to the intervention. As a result of our baseline study it is now known that even without the support of a nurse, 40% of epilepsy attendees do not revisit an ED in 12 months [10]. We included a TAU group to allow for this.

Secondly, a 2-session ENS intervention which lasts about 90 minutes in total may have been too brief to change self-management skills. More intensive interventions are used for those with other chronic, relapsing conditions. For type 1 diabetes, a 5-day course is used [45]. Indeed, it has been suggested that brief nurse interventions might serve to underline the label of epilepsy and adversely heighten a patient's awareness of epilepsy's restricting effects [41]. We though did not find any quantitative (or qualitative [46]) evidence to support this here, just as Bradley and Lindsday [47] did not in their Cochrane review of the effect of nurse interventions in the wider epilepsy population.

Finally, the ENSs tailored the content of intervention sessions to the needs of individual patients. This meant focus was not necessarily given to reducing participants' perceptions of stigma, their depression and anxiety, seizures, or to improving confidence in managing epilepsy. The results we have presented here on the predictors of subsequent ED by participants in our study, and also in-depth interviews with the participants themselves [48], suggest these may need to be addressed.

### Cost-effectiveness of the ENS intervention

Health economic evaluation provided a somewhat different perspective on the intervention's utility. Despite the additional input from the ENS, the cost of caring for an intervention group participant was on average £558 less. One reason for this was that the duration of hospital admissions following ED visits was shorter for the group who were offered the ENS intervention.

We used the EQ-5D health status questionnaire to calculate the QALY gain that occurred over follow-up associated with receiving the two treatments. The gain was slightly less for participants in ENS group than for those in the TAU group. This was unexpected, and runs counter to the finding that the more detailed and specific epilepsy QoL measure which did not show a difference between the two groups.

### Implications for future interventions

Why, after receiving the ENS intervention, participants stayed for shorter times in hospital following ED visits is unclear. Potentially the intervention led to a change in these patients' epilepsies that was not captured by our measures. For example, we did not assess seizure severity at follow-up. Future studies should consider this.

Previous models for ED use have focused on seizure frequency and only found a modest association between this single measure of disease severity and ED use [49]. Using multiple regression, we found that it was the degree to which patients felt stigmatized by epilepsy at baseline and, to a lesser extent, their confidence in managing epilepsy best predicted subsequent ED use. Our study is the first to provide longitudinal evidence on these variables' importance. This suggests felt stigma may be a driver of ED use and not simply a consequence [10].

How felt stigma and mastery influence ED use and what sort of intervention can modify them requires further investigation. We acknowledge that felt stigma may have emerged as a key predictor of ED use because it is a marker of disease severity, capturing more aspects of severity than other candidate variables. Indeed, the wider epilepsy literature consistently shows that felt stigma is associated with seizure frequency, epilepsy duration, number of AEDs, psychological distress and QoL. More complex statistical modelling by future studies could help further clarify felt stigma's role in ED use.

With regards optimization of the intervention, findings from interviews with our participants reported elsewhere [48] suggests that it might be appropriate for future interventions to systematically involve patients' significant others and offer them first aid training. Responsibility for patient care is often delegated to these persons when seizures occur.

### Limitations

Our study makes an important contribution to a small body of research. Its results though should be interpreted in light of some limitations.

Firstly, treatment allocation was not randomized. We did not receive funding to do this. Unknown baseline differences may have existed between our treatment groups, and this may reduce the accuracy of our treatment effect estimate. We did though seek to minimize the likelihood of differences by restricting recruitment to PWE from similar hospitals and areas. We also endeavoured to capture any differences by using a wide selection of baseline measures and adjusting for differences detected. We also used prospective recruitment. This can make estimates from non-randomized trials similar to those of randomized trials [50].

A second potential limitation is that 27% of those invited to participate agreed. It is now apparent that such rates are usual with ED attendees and in studies where serial assessment is required [45], [51]. Low acceptance does, nevertheless, raise the possibility that participants may not be representative. We have previously described how our participants' characteristics were generally comparable to those of nonparticipants [10].

The lower than anticipated uptake into the study by participants meant that our sample size was smaller than would have been preferred for the execution of our analysis plan. It meant that the case-variable ratio for our regression analyses was typically below the recommended 10 cases per variable rule [52]. For our adjusted ITT models, the median ratio was 6.6 (IQR 6.3–9.0). Consequently, some of our final regression models may be overfitted. It remains to be seen how well our findings are replicated by future, larger studies.

Thirdly, we recruited from an urban, ethnically diverse population with high social deprivation. Low educational attainment may reduce the ability of a brief intervention to influence outcome. It may also limit our results' generalizability to rural, less deprived populations. However, the potential similarity of our multi-ethnic population to those in metropolitan areas in the UK and beyond may make our evidence internationally generalizable. Further facilitating this is that the U.K. health service is publicly funded like those in most western countries.

At follow-up, we retained 81% of participants. This is favourable compared to previous studies [47], [53], [54]. Those lost were though more likely to have felt highly stigmatised at baseline and have been in the ENS treatment group. This may further limit the accuracy of our treatment estimate.

Finally, the researcher administering assessments was not blind to participants' treatment group. This may have influenced the assessments in some way, even though the same scoring procedures were followed for each participant, and the outcome negative.

## Conclusions

We tested an ENS-led intervention that aimed to reduce costly ED visits by PWE. A comparison to TAU alone, found no significant benefit on ED visits 6- and 12-months post-recruitment. No effects on the secondary psychosocial measures were also found. It did though slightly reduce total health care costs. This is the first study of an intervention for PWE who attend ED. Novel results from our analyses of long-term predictors of subsequent ED use suggest that to reduce visits, interventions should focus more on patients' perceptions of stigmatization due to epilepsy, and confidence managing epilepsy.

## Supporting Information

Table S1 **Baseline and outcome measures.**
(DOCX)

Table S2 **Baseline characteristics of study participants according to treatment group at follow-up assessments.**
(DOCX)

Table S3 **Efficacy analysis comparing treatment groups on primary and secondary outcome measures.**
(DOCX)
