# Correction: Clinical- and Cost-Effectiveness of a Nurse Led Self-Management Intervention to Reduce Emergency Visits by People with Epilepsy

**DOI:** 10.1371/journal.pone.0099913

**Published:** 2014-06-04

**Authors:** The PLOS ONE Staff

In the Author Contributions section, Leone Ridsdale (LR) should be listed as one of the persons who conceived and designed the experiments.
