# Supplementary material for: Clinical- and Cost-Effectiveness of a Nurse Led Self-Management Intervention to Reduce Emergency Visits by People with Epilepsy
Source: PLoS One. 2014 Mar 6;9(3):e90789. doi: 10.1371/journal.pone.0090789 (PMC3948384; doi:10.1371/journal.pone.0090789)
Supplement: Table S1 — Baseline and outcome measures. (DOCX) [file pone.0090789.s001.docx]

**Table S1** Baseline and outcome measures

| **Purpose** | **Assessments used in** | **Measure** | **Information** | **Range/ interpretation** |
| --- | --- | --- | --- | --- |
| ***Epilepsy characteristics*** | 1 | Medical records | Information collected on the participants’ epilepsies was restricted to that recorded in their medical records, which were coded using the ILAE’s 1989 classification system [1]. | - |
| ***Social deprivation*** | 1 | Index of Multiple Deprivation [2] | Deprivation was estimated by linking participants’ home postcodes to the Index of Multiple Deprivation | - |
| ***Emergency department & health service use*** | 1, 2, 3 | Client Services Receipt Inventory (CSRI) [3] | Examines use of health care services in the primary care setting (face-to-face general practice doctor consultations, telephone contacts, practice nurse contacts), secondary care services (inpatient, outpatient, day patient, emergency department), community health services (e.g. health visitors, district nurses, counselling or psychological therapists) and social care services (e.g. social workers, housing workers). Participants are asked to state whether they had used each service, how many contacts they had received and – where relevant – the average duration of service contact.  The CSRI also records: use of medication (names, doses, frequency and duration of use); receipt of informal care by asking how much extra help (in hours per week) they had received specifically because of their health problems (personal support, child care, help in/around the house, help outside the home, and other tasks); and for information on lost work days (for those in employment) due to health problems.  The CSRI asked participants about the previous 12 months for assessment 1 and previous 6 months for assessments 2 and 3. | Service costs were calculated by combining service use data with national unit cost information [4]. Medication costs were taken from routine Prescription Cost Analysis data [5]. |
| ***Seizure frequency*** | 1, 2, 3 | Frequency scale [6] | How many attacks have you had in the last 12 (assessment 1)/ 6 (assessment 2 and 3) months? | 0, 1, 2, 3, 4, 5, 6, 7, 8, 9, ≥10 |
| ***Seizure severity*** | 1, 2 | Liverpool Seizure Severity Scale 2.0 [7] | Patient rates “most severe seizure/s” in previous 4 weeks against 12 items concerning loss of consciousness, confusion, post-ictal sleepiness, time to recovery and injury. Linear transformation of sum of responses produces a total score. | Range 0 to100; higher= increasing severity. |
| ***Psychological distress*** | 1, 2, 3 | Hospital Anxiety and Depression Scale [8] | Patient rates experience of 7 Anxiety and 7 Depression symptoms in previous week. Symptoms of anxiety or depression relating also to physical disorders, such as headaches, insomnia, anergia and fatigue, are excluded. | Range 0 to 21 for each scale; higher score=more disturbance. For each scale, 8-10=borderline, ≥11= valid case [9]. |
| ***Quality of life*** | 1, 2, 3 | Quality of life in Epilepsy Inventory-10 [10] | 10-item measure of QoL in previous 4 weeks. Covers Epilepsy Effects (memory, physical and mental effects of AEDs), Mental Health (energy, depression) and Role Functioning (seizure worry, work, driving, social limits). | Range 10 to 50; higher= lower quality of life. |
| ***Felt stigma*** | 1, 2 | Stigma of Epilepsy Scale [11] | Patient asked to what extent, because of their epilepsy, they feel some people (i) are uncomfortable with them, (ii) treat them as inferior, (iii) would prefer to avoid them. Each item is responded to using Taylor et al.’s [12] scale: 0=“not at all”; 1=“yes, maybe”; 2=“yes, probably”; 3=“yes, definitely”. | Range 0 to 9; higher=more stigma; 0=no felt stigma; ≥1=stigmatized. |
| ***Generic health status*** | 1, 2, 3 | EuroQoL (EQ-5D) [13] | To generate quality-adjusted life years (QALYs) for cost-effectiveness analysis, participants completed the EQ-5D. Respondents create a “health status profile” by ticking boxes to indicate the degree of difficulty (1= no problem; 2= moderate problems; or 3= severe problems) they have on 5 dimensions on the day they are completing the questionnaire. The dimensions are mobility, self-care, usual activities, pain/discomfort, and anxiety/ depression. An example of a resulting health status profile is “1, 3, 1, 1, 3”. There are 243 theoretically possible states. | QALY score for each patient is calculated by determining the health-preference weight that his or her profile has been given by a representative UK sample when using the time-trade off method. Range -0.59 to 1; higher score= better perceived health state). [14] |
| ***Medication management*** | 1, 2, 3 | Epilepsy Self-Management Scale- Medication subscale [15] | Examines frequency, over the previous 12 (assessment 1)/ 6 (assessments 2 and 3) months, that patient performed behaviors associated with optimum adherence. Covers intentional and non-intentional nonadherence. Items rated on scale ranging from “never” to “always”. | Range 10-50; higher=better management. |
| ***Information satisfaction*** | 1, 2 | Satisfaction with Information about Medicines Scale [16] | 17-item scale asks patient to rate amount of, and satisfaction with, medication information received. Items 1 to 9 address action and usage of their respective AEDs and 10 to 17 concerns the potential problems of their medications. Items rated on scale ranging from 1=“None needed” to 5=“Too much”. | Range, post recording, 0-17; higher= more satisfied. |
| ***Epilepsy knowledge*** | 1, 2 | Epilepsy Knowledge Profile – General [17] | 55-item true/false questionnaire (34 Medical Knowledge items, 21 Social Knowledge items). Social Knowledge scale contains items on first aid for epilepsy. | Range Medical knowledge scale 0-35, Social Knowledge 0-21, higher= more knowledge. |
| ***Mastery*** | 1, 2, 3 | Epilepsy Mastery Scale [18] | Epilepsy specific 6-item adaptation of Pearlin and Schooner’s [19] internal vs. external locus of control measure. Patient rates extent to which they perceive their epilepsy as being under their control. Example of item: “Sometimes I feel helpless in dealing with my seizures”. Items rated on scale ranging from 1=“strongly agree” to 4=“strongly disagree”. | Range 6-24; higher= greater perceived mastery. |

**Notes** ED= emergency department; ENS= Epilepsy nurse specialist; Assessment 1= baseline; Assessment 2= 6-months post-recruitment; Assessment 3= 12-months post-recruitment

**References in Table S1**

1. Commission on Classification and Terminology of the International League Against Epilepsy (1989) Proposal for revised classification of epilepsies and epileptic syndromes. Epilepsia 30: 389-399.

2. Department for Communities and Local Government (2011) The English Indices of Deprivation 2010.

3. Beecham J, Knapp M (1992) Costing psychiatric interventions. In: Thornicroft G, Brewin C, Wing J, editors. Measuring Mental Health Needs London: Gaskell. pp. 163-183.

4. Curtis L (2010) Unit costs of health and social care. Canterbury: PSSRU.

5. The Health and Social Care Information Centre (2012) Prescription Cost Analysis England 2011. Health and Social Care Information Centre, Prescribing and Primary Care Services.

6. Thapar A, Kerr M, Harold G (2009) Stress, anxiety, depression, and epilepsy: Investigating the relationship between psychological factors and seizures. Epilepsy and Behavior 14: 134-140.

7. Scott-Lennox J, Bryant-Comstock L, Lennox R, Baker GA (2001) Reliability, validity and responsiveness of a revised scoring system for the Liverpool Seizure Severity Scale. Epilepsy Res 44: 53-63.

8. Zigmond AS, Snaith RP (1983) The hospital anxiety and depression scale. Acta Psychiatr Scand 67: 361-370.

9. Bjelland I, Dahl AA, Haug TT, Neckelmann D (2002) The validity of the Hospital Anxiety and Depression Scale. An updated literature review. J Psychosom Res 52: 69-77.

10. Cramer JA, Perrine K, Devinsky O, Meador K (1996) A brief questionnaire to screen for quality of life in epilepsy: the QOLIE-10. Epilepsia 37: 577-582.

11. Jacoby A (1994) Felt versus enacted stigma: a concept revisited: evidence from a study of people with epilepsy in remission. Social Science & Medicine 38: 269-274.

12. Taylor J, Baker GA, Jacoby A (2011) Levels of epilepsy stigma in an incident population and associated factors. Epilepsy Behav 21: 255-260.

13. Group TE (1990) EuroQol: a facility for the measurement of health related quality of life. Health Policy 16: 199-208.

14. Dolan P, Gudex C, Kind P, Williams A (1995) A social tariff for EuroQol: results from a UK population survey. York: University of York.

15. Dilorio C, Escoffery C, McCarty F, Yeager KA, Henry TR, et al. (2009 ) Evaluation of WebEase: an epilepsy self-management Web site. Health Education Research 24: 185-197.

16. Horne R, Hankins M, Jenkins R (2001) The Satisfaction with Information about Medicines Scale (SIMS): a new measurement tool for audit and research. Quality in Health Care 10: 135-140.

17. Jarvie S, Espie CA, Brodie MJ (1993) The development of a questionnaire to assess knowledge of epilepsy: 1--General knowledge of epilepsy. Seizure 2: 179-185.

18. Wagner AK, Keller SD, Kosinski M, Baker GA, Jacoby A, et al. (1995) Advances in methods for assessing the impact of epilepsy and antiepileptic drug therapy on patients' health-related quality of life. Qual Life Res 4: 115-134.

19. Pearlin LI, Schooler C (1978) The structure of coping. Journal of Health & Social Behavior 19: 2-21.
