# Supplementary material for: Clinical- and Cost-Effectiveness of a Nurse Led Self-Management Intervention to Reduce Emergency Visits by People with Epilepsy
Source: PLoS One. 2014 Mar 6;9(3):e90789. doi: 10.1371/journal.pone.0090789 (PMC3948384; doi:10.1371/journal.pone.0090789)
Supplement: Table S2 — Baseline characteristics of study participants according to treatment group at follow-up assessments. (DOCX) [file pone.0090789.s002.docx]

**Table S2** Baseline characteristics of study participants according to treatment group at follow-up assessments

| **Baseline measure (n/ %)** | **Treatment groups at assessment 2** | | | **Treatment groups at assessment 3** | | |
| --- | --- | --- | --- | --- | --- | --- |
|  | *TAU group (n=37)* | *ENS group (n=32)* | *OR (95% CI)* | *TAU group (n=37)* | *ENS group (n=32)* | *OR (95% CI)* |
| **Age at baseline** |  |  |  |  |  |  |
| 18-24 | 5 (13.5) | 5 (15.6) | 1.00 Reference | 5 (13.5) | 2 (6.3) | 1.00 Reference |
| 25-34 | 8 (21.6) | 8 (25.0) | 1.21 (0.39, 3.73) | 7 (18.9) | 9 (28.1) | 1.68 (0.54, 5.22) |
| 35-45 | 7 (18.9) | 5 (15.6) | 0.79 (0.22, 2.82) | 7 (18.9) | 6 (18.8) | 0.99 (0.29, 3.35) |
| 46-53 | 9 (24.3) | 6 (18.8) | 0.72 (0.22, 2.32) | 12 (32.4) | 6 (18.8) | 0.48 (0.16, 1.49) |
| 54-89 | 8 (21.6) | 8 (25.0) | 1.21 (0.39, 3.73) | 6 (16.2) | 0 (28.1) | 2.02 (0.63, 6.54) |
| **Gender** |  |  |  |  |  |  |
| Male | 20 (54.1) | 14 (43.8) | 1.00 Reference | 20 (54.1) | 13 (40.6) | 1.00 Reference |
| Female | 17 (45.9) | 18 (56.3) | 1.51 (0.58, 3.95) | 17 (45.9) | 19 (59.4) | 1.72 (0.66, 4.51) |
| **Ethnicity** |  |  |  |  |  |  |
| Other | 17 (45.9) | 10 (31.3) | 1.00 Reference | 23 (62.2) | 21 (65.6) | 1.00 Reference |
| White British | 20 (54.1) | 22 (68.8) | 0.54 (0.20, 1.45) | 14 (37.8) | 11 (34.4) | 0.86 (0.32, 2.33) |
| **Years of formal education** |  |  |  |  |  |  |
| 10 Least educated | 1 (2.7) | 1 (3.1) | 1.00 Reference | 2 (5.4) | 1 (3.1) | 1.00 Reference |
| 11 | 21 (56.8) | 13 (40.6) | 0.52 (0.20, 1.37) | 21 (56.8) | 12 (37.5) | 0.46 (0.17, 1.21) |
| 12 | 2 (5.4) | 2 (6.3) | 1.17 (0.15, 8.92) | 1 (2.7) | 2 (6.3) | 2.4 (0.20, 28.28) |
| 13-15.5 | 6 (16.2) | 6 (18.8) | 1.19 (0.34, 4.18) | 6 (16.2) | 7 (21.9) | 1.45 (0.43, 4.90) |
| 16-24 Most educated | 7 (18.9) | 10 (31.3) | 1.95 (0.64, 5.97) | 7 (18.9) | 10 (31.3) | 1.95 (0.64, 5.97) |
| **Deprivation score** |  |  |  |  |  |  |
| 13.97-22.70 Least deprived | 5 (13.5) | 11 (34.4) | 1.00 Reference | 4 (10.8) | 10 (31.3) | 1.00 Reference |
| 23.36-28.98 | 7 (18.9) | 3 (9.4) | 0.44 (0.10, 1.90) | 8 (21.6) | 3 (9.4) | 0.38 (0.09, 1.57) |
| 29.75-33.46 | 7 (18.9) | 5 (15.6) | 0.79 (0.22, 2.82) | 6 (16.2) | 7 (21.9) | 1.45 (0.43, 4.90) |
| 33.56-38.31 | 11 (297) | 7 (21.9) | 0.66 (0.22, 1.99) | 11 (29.7) | 6 (18.8) | 0.55 (0.17, 1.71) |
| 38.76-47.46 Most deprived | 7 (18.9) | 6 (18.8) | 0.99 (0.29, 3.35) | 8 (21.6) | 6 (18.8) | 0.84 (0.25, 2.76) |
| **Co-morbidity** |  |  |  |  |  |  |
| None | 20 (54.1) | 12 (37.5) | 1.00 Reference | 22 (59.5) | 11 (34.4) | 1.00 Reference |
| Psychiatric and/or medical | 17 (45.9) | 20 (62.5) | 1.96 (0.74, 5.18) | **15 (40.5)** | **21 (65.6)** | **2.8 (1.042, 7.52)** |
| **Years epilepsy diagnosed** |  |  |  |  |  |  |
| 2-4 | 4 (10.8) | 6 (18.8) | 1.00 Reference | 5 (13.5) | 7 (21.9) | 1.00 Reference |
| 5-8 | 9 (24.3) | 6 (18.8) | 0.72 (0.22, 2.32) | 7 (18.9) | 4 (12.5) | 0.61 (0.16, 2.34) |
| 9-15 | 7 (18.9) | 9 (28.1) | 1.68 (0.54, 5.22) | 7 (18.9) | 9 (28.1) | 1.68 (0.54, 5.22) |
| 16-34 | 7 (18.9) | 6 (18.8) | 0.99 (0.29, 3.35) | 9 (24.3) | 7 (21.9) | 0.87 (0.28, 2.71) |
| 35-67 | 10 (27.0) | 5 (15.6) | 0.50 (0.15,1.67) | 9 (24.3) | 5 (15.6) | 0.58 (0.17, 1.96) |
| **ED visits prior 12 months** |  |  |  |  |  |  |
| 1 | 14 (37.8) | 15 (46.9) | 1.00 Reference | 14 (37.8) | 14 (43.8) | 1.00 Reference |
| 2 | 12 (32.4) | 8 (25.0) | 0.69 (0.24, 2.01) | 11 (29.7) | 8 (25.0) | 0.79 (0.27, 2.31) |
| 3-4 | 3 (8.1) | 6 (18.8) | 2.62(0.59,11.58) | 2 (5.4) | 6 (18.8) | 4.04 (0.74, 21.91) |
| 5-25 | 8 (21.6) | 3 (9.4) | 0.38 (0.09, 1.57) | 10 (27.0) | 4 (12.5) | 0.39 (0.11, 1.39) |
| **Seizures prior 12 months** |  |  |  |  |  |  |
| 1-2 | 7 (18.9) | 7 (21.9) | 1.00 Reference | 5 (13.5) | 7 (21.9) | 1.00 Reference |
| 3-5 | 5 (13.5) | 8 (25.0) | 2.13 (0.61, 7.41) | 6 (16.2) | 8 (25.0) | 1.72 (0.52, 5.68) |
| 6-9 | 5 (13.5) | 6 (18.8) | 1.48 (0.40, 5.44) | 5 (13.5) | 7 (21.9) | 1.79 (0.50, 6.38) |
| 10 or more | 20 (54.1) | 11 (34.4) | 0.45 (0.17, 1.19) | **21 (56.8)** | **10 (31.3)** | **0.35 (0.13, 0.94)** |
| **Seizure severity score** |  |  |  |  |  |  |
| 0-5 Least severe | 11 (30.6) | 14 (43.8) | 1.00 Reference | 12 (32.4) | 15 (46.9) | 1.00 Reference |
| 7.5-50 | 10 (27.8) | 5 (15.6) | 0.48 (0.14, 1.61) | 10 (27.0) | 5 (15.6) | 0.50 (0.15, 1.67) |
| 52.5-67.5 | 7 (19.4) | 6 (18.8) | 0.96 (0.28, 3.24) | 9 (24.3) | 5 (15.6) | 0.58 (0.17, 1.96) |
| 70-90 Most severe | 8 (22.2) | 7 (21.9) | 0.98 (0.31, 3.12) | 6 (16.2) | 7 (21.9) | 1.45 (0.43, 4.90) |
| **Seizure onset** |  |  |  |  |  |  |
| Generalized or unknown | 17 (45.9) | 13 (40.6) | 1.00 Reference | 19 (51.4) | 12 (37.5) | 1.00 Reference |
| Focal | 20 (54.1) | 19 (59.4) | 1.24 (0.47, 3.26) | 18 (48.6) | 20 (62.5) | 1.76 (0.67, 4.64) |
| **AEDS prescribed** |  |  |  |  |  |  |
| 0 | 1 (2.7) | 0 (0.0) | 1.00 Reference | 1 (2.7) | 1 (3.1) | 1.00 Reference |
| 1 | 16 (43.2) | 19 (59.4) | 1.92 (0.73, 5.04) | 16 (43.2) | 29 (59.4) | 1.92 (0.73, 5.04) |
| 2 | 14 (37.8) | 10 (31.3) | 0.75 (0.27, 2.05) | 15 (40.5) | 10 (31.3) | 0.67 (0.25, 1.816) |
| 3-5 | 6 (16.2) | 3 (9.4) | 0.54 (0.12, 2.36) | 5 (13.5) | 2 (6.3) | 0.43 (0.08, 2.40) |
| **Depression score** |  |  |  |  |  |  |
| 0-1 Least symptoms | 9 (24.3) | 3 (9.4) | 1.00 Reference | 1 (3.1) | 11 (29.7) | 1.00 Reference |
| 2-3 | 11 (29.7) | 8 (25.0) | 0.79 (0.27, 2.31) | 19 (59.4) | 8 (21.6) | 1.02 (0.32, 3.22) |
| 4-5 | 4 (10.8) | 6 (18.8) | 1.90 (0.48, 7.53) | 0 (0.0) | 4 (10.8) | 1.90 (0.48, 7.53) |
| 6-7 | 5 (13.5) | 6 (18.8) | 1.48 (0.40, 5.44) | 10 (31.3) | 7 (18.9) | 1.20 (0.37, 3.92) |
| 8-19 Most symptoms | 8 (21.6) | 9 (28.1) | 1.42 (0.47, 4.29) | 2 (6.3) | 7 (18.9) | 1.68 (0.54, 5.22) |
| **Anxiety score** |  |  |  |  |  |  |
| 0-4 Least symptoms | 7 (18.9) | 5 (15.6) | 1.00 Reference | 7 (18.9) | 5 (15.6) | 1.00 Reference |
| 5-7 | 9 (24.3) | 10 (31.3) | 1.41 (0.49, 4.11) | 8 (21.6) | 10 (31.3) | 1.65 (0.55, 4.90) |
| 8-9 | 8 (21.6) | 3 (9.4) | 0.38 (0.09, 1.57) | 8 (21.6) | 3 (9.4) | 0.38 (0.09, 1.57) |
| 10-12 | 7 (18.9) | 8 (25.0) | 1.43 (0.45, 4.54) | 7 (18.9) | 8 (25.0) | 1.43 (0.45, 4.54) |
| 13-19 Most symptoms | 6 (16.2) | 6 (18.8) | 1.19 (0.34, 4.18) | 7 (18.9) | 6 (18.8) | 0.99 (0.29, 3.35) |
| **QOL score** |  |  |  |  |  |  |
| 13-18 Highest QoL | 8 (21.6) | 4 (12.5) | 1.00 Reference | 8 (21.6) | 4 (12.5) | 1.00 Reference |
| 19-23 | 7 (18.9) | 9 (28.1) | 1.68 (0.54, 5.22) | 7 (18.9) | 8 (25.0) | 1.43 (0.45, 4.54) |
| 24-26 | 4 (10.8) | 5 (15.6) | 1.53 (0.37, 6.32) | 5 (13.5) | 6 (18.8) | 1.48 (0.40, 5.44) |
| 27-33 | 10 (27.0) | 6 (18.8) | 0.62 (0.19, 1.98) | 10 (27.0) | 6 (18.8) | 0.62 (0.20, 1.98) |
| 34-36 Lowest QoL | 8 (21.6) | 8 (25.0) | 1.21 (0.39, 3.73) | 7 (18.9) | 8 (25.0) | 1.43 (0.45, 4.54) |
| **Felt stigma score** |  |  |  |  |  |  |
| 0 Least stigma | 11 (29.7) | 13 (40.6) | 1.00 Reference | 10 (27.0) | 13 (40.6) | 1.00 Reference |
| 1-2 | 8 (21.6) | 7 (21.9) | 1.02 (0.32, 3.22) | 9 (24.3) | 7 (21.9) | 0.87 (0.28, 2.71) |
| 3-4 | 6 (16.2) | 7 (21.9) | 0.63 (0.24, 1.67) | 7 (18.9) | 9 (28.1) | 1.68 (0.54, 5.22) |
| 5-9 Most stigma | 12 (32.4) | 5 (15.6) | 0.39 (0.12, 1.26) | **11 (29.7)** | **3 (9.4)** | **0.25 (0.06, 0.98)** |
| **Medication management** |  |  |  |  |  |  |
| 13-39 Lowest skills | 4 (11.1) | 7 (21.9) | 1.00 Reference | 5 (13.9) | 6 (18.8) | 1.00 Reference |
| 40-44 | 6 (16.7) | 8 (25.0) | 1.89 (0.62, 5.79) | 4 (11.1) | 8 (25.0) | 2.96 (0.85, 10.32) |
| 45-46 | 8 (22.2) | 7 (21.9) | 1.17 (0.40, 3.42) | 7 (19.4) | 8 (25.0) | 1.59 (0.54, 4.67) |
| 47-48 | 6 (16.7) | 5 (15.6) | 1.15 (0.35, 3.80) | 8 (22.2) | 6 (18.8) | 0.99 (0.33, 2.99) |
| 49-50 Highest skills | 12 (33.3) | 5 (15.6) | 0.49 (0.16, 1.53) | 12 (33.3) | 4 (12.5) | 0.40 (0.12, 1.33) |
| **Satisfaction info** |  |  |  |  |  |  |
| 1-4 Least satisfied | 6 (16.7) | 5 (16.1) | 1.00 Reference | 6 (16.7) | 5 (16.1) | 1.00 Reference |
| 5-7 | 8 (22.2) | 6 (19.4) | 0.84 (0.25, 2.78) | 8 (22.2) | 6 (19.4) | 0.84 (0.25, 2.78) |
| 8-9 | 5 (13.9) | 7 (22.6) | 1.81 (0.51, 6.47) | 4 (11.1) | 8 (25.8) | 2.78 (0.74, 10.46) |
| 10-11 | 8 (22.2) | 6 (19.4) | 0.84 (0.25, 2.78) | 7 (19.4) | 6 (19.4) | 0.99 (0.29, 3.38) |
| 12-17 Most satisfied | 9 (25.0) | 7 (22.6) | 0.88 (0.28, 2.73) | 11 (30.6) | 6 (19.4) | 0.55 (0.17, 1.72) |
| **Social knowledge** |  |  |  |  |  |  |
| 8-12 Lowest knowledge | 9 (24.3) | 3 (9.4) | 1.00 Reference | 9 (24.3) | 4 (12.5) | 1.00 Reference |
| 13-14 | 10 (27.0) | 9 (28.1) | 1.06 (0.36, 3.07) | 11 (29.7) | 8 (25.0) | 0.79 (0.27, 2.31) |
| 15-15 | 9 (24.3) | 9 (28.1) | 1.22 (0.41, 3.60) | 9 (24.3) | 8 (25.0) | 1.04 (0.34, 3.13) |
| 16-20 Highest knowledge | 9 (24.3) | 11 (34.4) | 1.63 (0.57, 4.68) | 8 (21.6) | 12 (37.5) | 2.18 (0.75, 6.33) |
| **Medical knowledge** |  |  |  |  |  |  |
| 15-21 Lowest knowledge | 9 (24.3) | 4 (12.5) | 1.00 Reference | 9 (24.3) | 5 (15.6) | 1.00 Reference |
| 22-24 | 9 (24.3) | 7 (21.9) | 0.87 (0.28, 2.71) | 9 (24.3) | 7 (21.9) | 0.87 (0.28, 2.71) |
| 25-26 | 6 (16.2) | 4 (12.5) | 0.74 (0.19, 2.92) | 7 (18.9) | 4 (12.5) | 0.61 (0.16, 2.34) |
| 27-28 | 6 (16.2) | 10 (31.3) | 2.35 (0.74, 7.48) | 5 (13.5) | 9 (28.1) | 2.50 (0.74, 8.54) |
| 29-32 Highest knowledge | 7 (18.9) | 7 (21.9) | 1.20 (0.37, 3.92) | 7 (18.9) | 7 (21.9) | 1.20 (0.37, 3.92) |
| **Mastery** |  |  |  |  |  |  |
| 6-12 Lowest confidence | 10 (27.0) | 7 (21.9) | 1.00 Reference | 10 (27.0) | 7 (21.9) | 1.00 Reference |
| 13-14 | 7 (18.9) | 7 (21.9) | 1.20 (0.37, 3.92) | 6 (16.2) | 8 (25.0) | 1.72 (0.52, 5.68) |
| 15-15 | 5 (13.5) | 7 (21.9) | 1.79 (0.50, 6.38) | 4 (10.8) | 7 (21.9) | 2.31 (0.60, 8.85) |
| 16-17 | 6 (16.2) | 5 (15.6) | 0.96 (0.26, 3.52) | 8 (21.6) | 5 (15.6) | 0.67 (0.19, 2.33) |
| 18-21 Highest confidence | 9 (24.3) | 6 (18.8) | 0.72 (0.22, 2.32) | 9 (24.3) | 5 (15.6) | 0.58 (0.17, 1.96) |

**Notes** AED= antiepileptic drug; CI= Confidence interval; ED= Emergency department; ENS= Epilepsy Nurse Specialist; OR= Odds-ratio; Primary care QoF 8 score= Quality and Outcomes Framework;; percentage of people with epilepsy (aged ≥16) prescribed AEDs in the local population who were seizure free in the previous 12 months as recorded by primary care medical practices in England in 2009/10; QoL= Quality of Life; TAU= Treatment as usual.

P<0.10 shown in **bold**; Logistic regression used.
