# Supplementary material for: Clinical- and Cost-Effectiveness of a Nurse Led Self-Management Intervention to Reduce Emergency Visits by People with Epilepsy
Source: PLoS One. 2014 Mar 6;9(3):e90789. doi: 10.1371/journal.pone.0090789 (PMC3948384; doi:10.1371/journal.pone.0090789)
Supplement: Table S3 — Efficacy analysis comparing treatment groups on primary and secondary outcome measures. (DOCX) [file pone.0090789.s003.docx]

# Table S3 Efficacy analysis comparing treatment groups on primary and secondary outcome measures

| **Outcome measure** | **Assessment 2 (n=65)** | | **Assessment 3 (n=67)** | |
| --- | --- | --- | --- | --- |
|  | *Unadjusted*  *IRR/ Coefficient (95% CI)* | *Adjusted*  *IRR/ Coefficient (95% CI)* | *Unadjusted*  *IRR/ Coefficient (95% CI)* | *Adjusted*  *IRR/ Coefficient (95% CI)* |
| **Primary outcome measure** |  |  |  |  |
| Emergency department visits | 0.89 (0.37, 2.16) | 1.72 (0.86, 3.43) | 0.46 (0.17, 1.25) | 2.24 (0.73, 6.85) |
| **Secondary outcome measure** |  |  |  |  |
| Quality of Life (higher= poorer quality) | 1.57 (-2.25, 5.40) | 1.05 (-1.40, 3.50) | 2.48 (-1.35, 6.31) | 3.08 (-0.73, 6.89) |
| Seizure frequency (higher= more seizures) | -0.29 (-2.43, 1.83) | -0.18 (-1.82, 1.47) | -0.33 (-2.30, 1.64) | 0.49 (-1.06, 2.04) |
| Anxiety (higher= more symptoms) | -0.28 (-2.63, 2.07) | -0.97 (-2.61, 0.67) | -1.12 (-3.42, 1.17) | -1.57 (-3.54, 0.40) |
| Depression (higher= more symptoms) | 0.30 (-1.71, 2.32) | -0.55 (-1.88, 0.78) | 0.09 (-1.86, 2.03) | -1.79 (-3.83, 0.25) |
| Medication management skills (higher= better skills) | 2.70 (-5.74, 11.13) | 2.68 (-5.11, 10.46) | -0.03 (-3.24, 3.18) | 1.88 (-2.70, 6.47) |
| Mastery (higher= greater confidence) | -0.70 (-2.40, 0.99) | -0.97 (-2.34, 0.40) | 0.30 (-1.40, 2.00) | 1.32 (-2.75, 5.38) |
| Epilepsy social knowledge (higher= more knowledgeable) | -0.24 (-1.58, 1.11) | -0.77 (-1.87, 0.34) | - | - |
| Epilepsy medical knowledge (higher= more knowledgeable) | 0.68 (-1.37, 2.74) | -0.73 (-2.17, 0.71) | - | - |
| Felt stigma (higher= more stigmatization) | -1.01 (-2.61, 0.59) | -0.24 (-1.26, 0.78) | - | - |
| Satisfaction with medication information (higher= more satisfied) | 0.22 (-2.05, 2.50) | 0.09 (-1.70, 1.88) | - | - |

**Notes** IRR= incidence rate-ratio; CI= confidence interval; AED = antiepileptic drug; ED Emergency Departments

P<0.05 shown in **bold**; Negative binomial regression used for outcome measure emergency visits and linear regression used for all remaining measures.

IRRs less than 1 here represent a lower visit rate in the ENS treatment group relative to TAU, whilst IRRs greater than 1, indicate a higher rate.

For secondary outcome measures, positive coefficients here indicate an increase in the score on the outcome variable associated with receiving the ENS led self-management intervention, whilst a negative coefficient the opposite.

Adjustments were made for baseline variables related to outcome at P<0.10:

Emergency department (ED) visits: Baseline Seizure frequency (assessment 3) ED visits (assessments 2, 3), Seizure severity (3), AED number (2,3), Depression (2,3), Anxiety (2,3), Quality of Life (QoL) (3), Felt stigma (3), Medical knowledge (3), Mastery (2,3). Number of covariates in final assessment 2 model= 5; Number of covariates in final assessment 3 model= 10.

QoL: Baseline Seizure frequency (2,3), ED visits (2,3), AED number (2), Depression (2,3), Anxiety (2,3), QoL (2,3), Stigma (2,3), Satisfaction medication information (2), Social knowledge (3), Medical knowledge (3), Mastery (2,3). Number of covariates in final assessment 2 model= 9; Number of covariates in final assessment 3 model= 9.

Seizure frequency: Baseline Seizure frequency (2,3), Primary care seizure-free rate (QOF score 8) (3), Gender (2), ED visits (2,3), Seizure severity (2), AED number (2,3), Depression (2,3), Anxiety (2,3), QoL (2,3), Felt stigma (2,3), Medication management (2), Social knowledge (3), Mastery (2,3). Number of covariates in final assessment 2 model= 11; Number of covariates in final assessment 3 model= 10.

Anxiety: Baseline Seizure frequency (3), ED visits (2,3), AED number (2), Depression (2,3), Anxiety (2,3), QoL (2,3), Felt stigma (2,3), Social knowledge (3), Mastery (2,3). Number of covariates in final assessment 2 model= 7; Number of covariates in final assessment 3 model= 8.

Depression: Baseline Age (3), Education (3), Deprivation (3), ED visits (2,3), Depression (2,3), Anxiety (2,3), QoL (2,3), Felt stigma (2,3), Social knowledge (3), Medical knowledge (3), Satisfaction with medication information (2), Mastery (2,3). Number of covariates in final assessment 2 model= 7; Number of covariates in final assessment 3 model= 11.

Medication Management Skills: Baseline Age (2), Sex (2), Epilepsy duration (2), AED number (3), Depression (3), Medication Management (3), Medical knowledge (3). Number of covariates in final assessment 2 model= 3; Number of covariates in final assessment 3 model= 4.

Mastery: Baseline Seizure frequency (2,3), Gender (2), Ethnicity (3), Deprivation (3), ED visits (2,3), Seizure severity (2,3), AED number (2,3), Depression (2,3), Anxiety (2,3), QoL (2,3), Felt stigma (2,3), Social knowledge (3), Medical knowledge (3), Mastery (2,3). Number of covariates in final assessment 2 model= 10; Number of covariates in final assessment 3 model= 13.

Epilepsy social knowledge: Baseline Age, Education, Deprivation, Medication management skills, Social knowledge, Medical knowledge. Number of covariates in final assessment 2 model= 6.

Epilepsy medical knowledge: Baseline Age, Education, Deprivation, ED visits, Depression, Anxiety, Felt stigma, Social knowledge, Medical knowledge, Mastery. Number of covariates in final assessment 2 model= 10.

Felt stigma: Baseline Seizure frequency, Ethnicity, Deprivation, ED visits, Seizure severity, AED number, Depression, QoL, Felt stigma, Mastery. Number of covariates in final assessment 2 model= 10.

Satisfaction with medication information: Baseline Primary care QOF 8 score, Deprivation, ED visits, Depression, Anxiety, QoL, Felt stigma, Satisfaction with medication information, Medical knowledge, Mastery. Number of covariates in final assessment 2 model= 10.
